# Identification of causative variants in *TXNL4A* in Burn-McKeown syndrome and isolated choanal atresia

**DOI:** 10.1038/ejhg.2017.107

**Published:** 2017-07-26

**Authors:** Jacqueline A C Goos, Sigrid M A Swagemakers, Stephen R F Twigg, Marieke F van Dooren, A Jeannette M Hoogeboom, Christian Beetz, Sven Günther, Frank J Magielsen, Charlotte W Ockeloen, Maria A Ramos-Arroyo, Rolph Pfundt, Helger G Yntema, Peter J van der Spek, Philip Stanier, Dagmar Wieczorek, Andrew O M Wilkie, Ans M W van den Ouweland, Irene M J Mathijssen, Jane A Hurst

**Affiliations:** 1 Department of Plastic and Reconstructive Surgery and Hand Surgery, Erasmus MC, University Medical Center Rotterdam, Rotterdam, The Netherlands; 2 Department of Bioinformatics, Erasmus MC, University Medical Center Rotterdam, Rotterdam, The Netherlands; 3 Clinical Genetics Group, Weatherall Institute of Molecular Medicine, University of Oxford, John Radcliffe Hospital, Oxford, UK; 4 Department of Clinical Genetics, Erasmus MC, University Medical Center Rotterdam, Rotterdam, The Netherlands; 5 Department of Clinical Chemistry and Laboratory Medicine, Jena University Hospital, Jena, Germany; 6 Department of Human Genetics, Radboud University Medical Center, Nijmegen, The Netherlands; 7 Department of Medical Genetics, Complejo Hospitalario de Navarra, IdiSNA, Navarra Institute for Health Research, Pamplona, Navarra, Spain; 8 Genetics and Genomic Medicine, UCL Institute of Child Health, London, UK; 9 Institute of Human Genetics, Heinrich-Heine-University, Medical Faculty, Düsseldorf, Germany; 10 Clinical Genetics Department, NE Thames Genetics Service, Great Ormond Street Hospital, London, UK

## Abstract

Burn-McKeown syndrome (BMKS) is a rare syndrome characterized by choanal atresia, prominent ears, abnormalities of the outer third of the lower eyelid, structural cardiac abnormalities, conductive and sensorineural hearing loss, and cleft lip. Recently, causative compound heterozygous variants were identified in *TXNL4A*. We analyzed an individual with clinical features of BMKS and her parents by whole-genome sequencing and identified compound heterozygous variants in *TXNL4A* (a novel splice site variant (c.258-2A>G, (p.?)) and a 34 bp promoter deletion (hg19 chr18:g.77748581_77748614del (type 1Δ) in the proband). Subsequently, we tested a cohort of 19 individuals with (mild) features of BMKS and 17 individuals with isolated choanal atresia for causative variants in *TXNL4A* by dideoxy-sequence analysis. In one individual with BMKS unrelated to the first family, we identified the identical compound heterozygous variants. In an individual with isolated choanal atresia, we found homozygosity for the same type 1Δ promoter deletion, whilst in two cousins from a family with choanal atresia and other minor anomalies we found homozygosity for a different deletion within the promoter (hg19 chr18: g.77748604_77748637del (type 2Δ)). Hence, we identified causative recessive variants in *TXNL4A* in two individuals with BMKS as well as in three individuals (from two families) with isolated choanal atresia.

## Introduction

Burn-McKeown syndrome (BMKS) was first described in five children by Burn *et al.*^1^ It is a rare disorder characterized by choanal atresia, prominent ears, hypertelorism with short palpebral fissures and abnormalities of the outer third of the lower eyelids. Other features that can be observed are structural cardiac abnormalities, conductive and sensorineural hearing loss, and unilateral cleft lip.^1^ There is clinical overlap with both Treacher Collins and CHARGE syndromes.

Burn *et al*^1^ advised counseling a high recurrence risk in families with BMKS, but the inheritance pattern remained unclear until recently. As the syndrome was repeatedly described in siblings and mainly in males, an autosomal recessive^1^ or X-linked inheritance pattern was suggested.^2^ However, the identification of a chromosomal rearrangement, 46,XX,r(18)(p14q23), in an isolated female case with features of BMKS suggested terminal 18p or 18q as the locus for the disorder.^1^ This was borne out by the detection of causative compound heterozygous variants in *TXNL4A* (located on chromosome 18q23), identified more than 20 years after the initial description of BMKS.^3^

*TXNL4A* is a member of the U5 spliceosomal complex that is critical for pre-mRNA splicing.^4^ It has been suggested that reduced expression of *TXNL4A* influences the splicing of a specific subset of pre-mRNAs, resulting in the tissue-specific phenotypic spectrum of BMKS.^3, 5^ In the present study, we describe new causative variants in *TXNL4A* and expand the associated phenotypic spectrum.

## Subjects and methods

### Subjects

Ethical approval from the board of the Medical Ethical Committee of the Erasmus MC, University Medical Center Rotterdam, the Netherlands, was given for whole-genome sequencing (WGS; MEC-2012-140) and for retrospective data collection (MEC-2013-547). UK samples were collected following approval from the Great Ormond Street Hospital for Children NHS Trust Ethics Committee (REC No. 08H0713/46). Informed consent was received from the individuals and parents.

**Figure 1:**
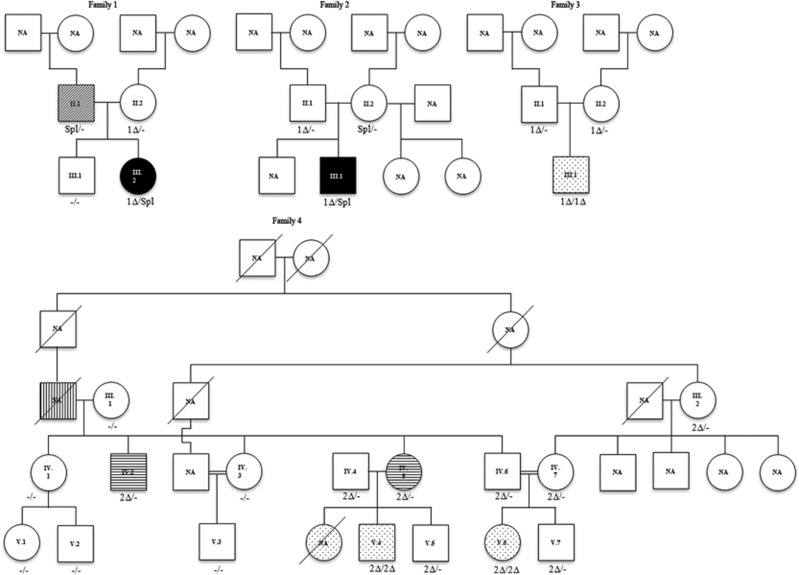
Pedigrees of families. Symbol definitions: 
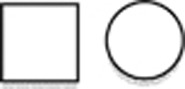
 not affected, 
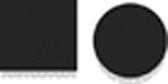
 BMKS, 
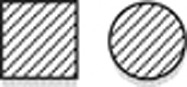
 lagophtalmos, 
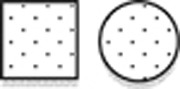
 choanal atresia, 
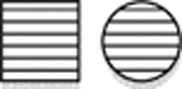
 prognathism and 
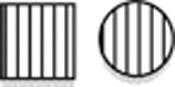
 maxillary hypoplasia. 1Δ, promoter deletion type 1Δ (hg19 chr18:g.77748581_77748614del), 2Δ, promoter deletion type 2Δ (hg19 chr18: g.77748604_77748637del); NA, not available; Spl, splice site variant, −/−, no causative variant in *TXNL4A*.

WGS was performed on DNA of the members of family 1 (see Figure 1).

To identify further mutation-positive individuals, dideoxy-sequence analysis of the promoter region, the exons and the exon/intron boundaries of *TXNL4A* was performed using DNA of 19 individuals who have clinical features that overlap with (mild) BMKS. In addition, 17 individuals with isolated choanal atresia were also tested for mutations in *TXNL4A.* In addition, 29 of the DNA samples were analyzed for copy number variations in *TXNL4A* using a multiplex ligation-dependent probe amplification (MLPA) assay as described by Wieczorek *et al.*^3^

The variants identified were described according to HGVS nomenclature,^6^ using the reference sequences NM_006701.2, ENST00000269601 and ENSG00000141759, on GRCh37/hg19 and were submitted to the Leiden Open Variation Database (http://www.lovd.nl/TXNL4A).

### Whole-genome sequencing

Before publication of the paper of Wieczorek *et al*,^3^ WGS was performed on DNA of the proband of family 1 (III.2) and her parents (see Figure 1) by Complete Genomics, a BGI company (Mountain View, CA, USA) as described by Drmanac.^7^ Variants were annotated using NCBI build 36.3/hg18 and dbSNP build 130. Data were analyzed using cga tools version 1.6.0.43 and TIBCO Spotfire 7.0.0 (TIBCO Software Inc., Boston, MA, USA). The annotations were converted to GRCh37/hg19 by using Human BLAT search on the UCSC website (Kent Informatics, Inc., Santa Cruz, CA, USA) as described previously.^8^

An autosomal dominant disease model was tested in family 1. The analysis was restricted to novel non-synonymous variants, variants disrupting a splice site (±two bp), and insertions or deletions in the coding sequence (±50 bp).

The remaining variants were analyzed with Annovar,^9^ to get an indication of the pathogenicity and the ESP frequency as given in Exome Variant Server (EVS, NHLBI GO Exome Sequencing Project (ESP), Seattle, WA (URL: http://evs.gs.washington.edu/EVS/) [April 2014 accessed]). We focused on variants that were located in genes without loss-of-function mutations in EVS.

### Confirmation by dideoxy-sequence analysis

The variants identified by WGS were validated by dideoxy-sequence analysis (all primer sequences are provided in Supplementary Table 1). Amplification reactions were performed according to standard procedures. PCR products were purified using Agencourt AMPure (Agencourt, Beckman Coulter Inc., Brea, CA, USA). Direct sequencing of both strands was performed using Big Dye terminator version 3.1 (Applied Biosystems, Foster City, CA, USA) as recommended by the manufacturer. Dye terminators were removed using Agencourt CleanSeQ (Agencourt) and loaded on an ABI 3130XL Genetic Analyzer (Applied Biosystems). The sequences were analyzed using SeqPilot version 4.1.2 build 507 (JSI Medical Systems Gmbh, Kippenheim, Germany).

### Confirmation of effect of the variants on RNA expression

To analyze the effect of the variants on RNA expression, cDNA was analyzed by restriction enzyme digestion and deep sequencing. First, RNA was extracted according to the manufacturer’s protocol from venous blood collected into PAXgene Blood RNA tubes (Qiagen N.V., Venlo, The Netherlands) from individuals II.1, II.2 and III.2 (Figure 1, family 1). cDNA was synthesized using the RevertAid First Strand cDNA kit (Thermo Scientific Inc., Waltham, MA, USA), with random hexamer primers according to the manufacturer’s instructions. Primers were designed for all exons and intron two to screen for truncations and intron retention (Supplementary Table 1). cDNA was amplified and products were electrophoresed on agarose gels and DNA was visualized by staining with ethidium bromide (EtBr).

### Digestion with PshAI and AhdI and electrophoresis of digests

To assess the effect of the paternal variant on RNA expression in family 1, cDNA amplification products were digested with PshAI or AhdI (New England Biolabs Inc., Ipswich, MA, USA) and analyzed by agarose gel electrophoresis. The forward primer was mutated to allow digestion with AhdI (underlined and bold ‘a’ in Supplementary Table 1).

### Deep sequencing by Ion PGM Sequencing

To distinguish between the wild-type and mutant allele, two heterozygous SNPs in the 3'-UTR of *TXNL4A* that were identified in the WGS data from the parents of the family 1 proband were used. To quantify the effect of the maternal variant, deep sequencing covering the SNP was performed on cDNA of the proband. Primers are given in Supplementary Table 1. The cDNA amplification products were diluted 1/100 and 2 μl was used in a second round PCR, with a reverse primer including the Ion PGM P1 adapter, and a forward primer with the Ion PGM A adapter sequence and a barcode sequence (reaction conditions available on request). Amplification products (roughly equal amounts as judged by EtBr staining by agarose gel electrophoresis) were combined and then purified with AMPure beads (Beckman Coulter). Emulsion PCR and enrichment was performed with the Ion PGM Template OT2 200 Kit (Life Technologies) according to the manufacturer’s instructions. Sequencing of enriched templates was performed on the Ion Torrent PGM (Life Technologies) for 125 cycles using the Ion PGM Sequencing 200 kit v2 and Ion 316 chips. Data were processed with Ion Torrent platform-specific pipeline software v4.2.1.

## Results

### Whole-genome sequencing of family 1

**Figure 2:**
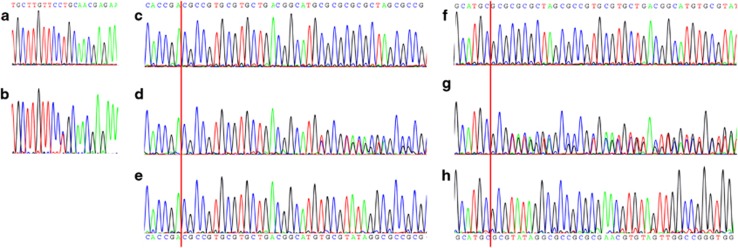
Electropherograms of dideoxy-sequence analyses. Red line indicates the start of the deletion. (**a**) control DNA. (**b**) splice site variant in DNA of III.2 of family 1. (**c**) region of type 1Δ deletion in control DNA. (**d**) heterozygous type 1Δ deletion in DNA of III.2 of family 1. (**e**) homozygous type 1Δ deletion as seen in DNA of III.1 of family 3. (**f**) region of type 2Δ deletion in control DNA. (**g**) heterozygous type 2Δ deletion as seen in DNA of IV.6 of family 4. (**h**) homozygous type 2Δ deletion as seen in DNA of V.4 of family 4.

The proband of family 1 and her parents were analyzed by WGS. Variants remaining after the various filtering steps are available on request. By testing the expected autosomal dominant disease model, we identified a novel splice site variant upstream of the last exon in *TXNL4A* (c.258-2A>G, (p.?)), that was inherited from the father. According to both cga tools and ANNOVAR, the splice site variant was predicted to be deleterious. As this variant was present in the 3′ splice site consensus sequence, it was highly conserved with a PhyloP score of 1.824 × 10^−5^. The variant was not present in ESP, 1000 Genomes (The 1000 Genomes Project Consortium, 2012, URL: http://www.1000genomes.org/^10^), Ensembl,^11^ nor in our in-house Huvariome database.^12^ However, it did have an allele frequency of 7.31 × 10^−6^ in gnomAD.^13^ During the analysis, the phenotype was recognized as suspected BMKS. Therefore, recessive variants on chromosome 18 were implicated and the DNA sequence of *TXNL4A* was scrutinized manually. Based on the publication of Wieczorek *et al*,^3^ the likely significance of a 34 bp deletion within the promoter became apparent (hg19 chr18:g.77748581_77748614del, referred to as type 1Δ and inherited from the mother).^3^ The presence of both variants was confirmed by dideoxy sequencing (Figure 2).

### cDNA analysis of *TXNL4A*

RT-PCR analysis of the proband of family 1 and her parents, followed by agarose gel electrophoresis, did not show any aberrantly spliced products in any of the samples, despite the presence of the predicted splice site variant in both the proband and her father. WGS data indicated that both parents each carried a different sequence change within the 3′ UTR of *TXNL4A* that was not inherited by their daughter (thus located on the reference alleles): the father was heterozygous for hg19 chr18:g.77733297dupC (rs77355432, dbSNP build 146) and the mother for hg19 chr18:g.77733273C>T (rs4798931). Primers were designed to amplify both SNPs in one amplicon.

**Figure 3:**
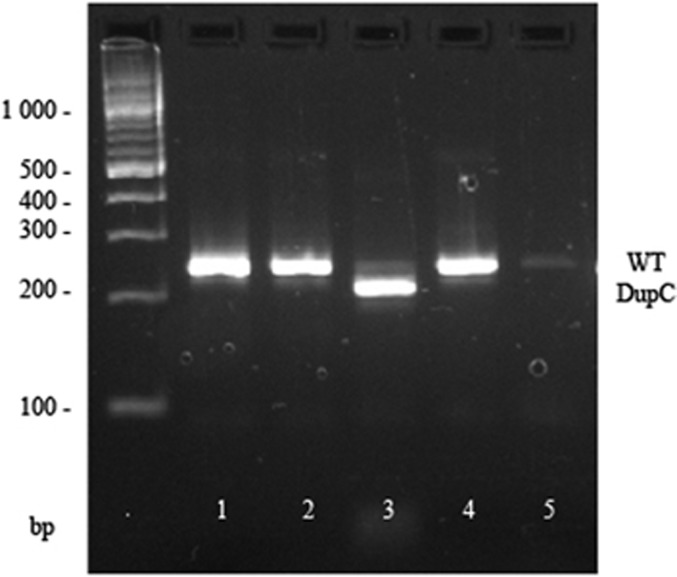
Restriction enzyme digest analysis of rs77355432 SNP in cDNA from family 1; expected fragment sizes reference allele 249 bp (WT), alternative allele 215+34 bp (dupC). Lane 1, uncut DNA. Lane 2, cDNA from the proband is not digested with AhdI. Lane 3, cDNA of the father is cut almost to completion with AhdI. Lane 4, cDNA of the mother is uncut with AhdI. Lane 5, negative control.

The effect of the 3′ acceptor variant c.258-2A>G on splicing, was assessed using the restriction enzymes AhdI and PshAI. AhdI was specific for the 3′ UTR dupC (reference allele, not passed to the proband), while PshAI was specific for the mutant allele in cDNA from the father (passed to the proband). The primer was designed to amplify the alleles equally, had they both been present in the cDNA. Using AhdI, the cDNA of the father was digested almost to completion (Figure 3), and using PshAI the cDNA remained undigested (data not shown), indicating that there was no or extremely reduced mature transcript from the mutant allele.

Deep sequencing of maternal cDNA demonstrated reduced expression from the allele with the type 1Δ: in a total of 36 924 reads, 8 636 reads (23%) were of the reference C (the allele with the promoter variant), and 28 283 reads (77%) contained the variant T (wildtype promoter allele).

### Expansion of the mutation spectrum

Dideoxy sequencing of *TXNL4A* was performed on DNA obtained from 19 index cases with (mild) features of BMKS. The proband of family 2 (Figure 1, III.1 of family 2) was found to carry the identical variants to those of the family 1 proband. Analysis of parental samples showed that they were present in compound heterozygous state with the type 1Δ deletion inherited from the father and the splice site variant inherited from the mother. In addition, 17 index cases with choanal atresia were tested for mutations in *TXNL4A*. In two probands, homozygosity for a promoter deletion was identified. The proband of family 3 (Figure 1, III.1 of family 3) had a uniparental disomy of chromosome 18p11.32q23, leading to homozygosity for the type 1Δ deletion. The family 4 proband (Figure 1, V.4 of family 4) was homozygous for a slightly different promoter deletion: hg19 chr18: g.77748604_77748637del corresponding to type 2Δ in Wieczorek *et al.*^3^ The same genotype was also present in his first cousin (Figure 1, V.6 of family 4).

MLPA analysis was performed on the 29 samples with sufficient DNA available. This included samples of both families 3 and 4 (Table 2). Large rearrangements were not identified in any of these samples.

### Subjects

Nineteen individuals with clinical features overlapping with BMKS syndrome and 17 individuals with isolated choanal atresia were tested for mutations in *TXNL4A.* Causative variants were identified in five individuals. An overview of the clinical phenotype of these individuals is given in Table 1. The proband of family 4 and his first cousin (Figure 1, V.4 and V.6 of family 4) were previously described as patients 1 and 3 in the paper of Ramos-Arroyo *et al.*^14^ All individuals had choanal atresia and normal development. In three out of five, their pregnancy was complicated by polyhydramnios. The same three had a prominent nasal bridge. Maxillary hypoplasia was also seen in the same three individuals, and micrognathia was seen in two out of them. Two out of five individuals had defects of the lower eyelids. The proband of family 4 and his first cousin had oligodontia, however, due to the high prevalence of this condition,^15^ we cannot rule out that this is a separate condition that is segregating within this family.^14^

## Discussion

In two out of 20 unrelated individuals with clinical features of BMKS, we identified compound heterozygosity in *TXNL4A* for a novel splice site variant, c.258-2A>G, (p.?) and the type 1Δ promoter variant, hg19 chr18:g.77748581_77748614del. Our findings support the idea that causative *TXNL4A* variants do not completely ablate function in BMKS.^3^

In nine out of 11 affected families, Wieczorek *et al*^3^ found a rare loss-of-function variant (nonsense, frameshift or microdeletion) on one allele in conjunction with a promoter deletion on the other allele. In our deep sequencing analysis of RNA isolated from blood, expression of the type 1Δ mutant allele was reduced by ~69%. This is in keeping with a luciferase reporter gene assay, where the promoter activity of constructs containing type 1Δ deletion was reduced by 59% compared to a wild-type construct.^3^ The presence of the splice-site variant on the other *TXNL4A* allele of the family 1 proband most likely leads to loss-of-function, since analysis of paternal cDNA based on the closely adjacent SNP rs77355432, indicated no evidence of transcription of this allele (Figure 3). Wieczorek *et al*^3^ have shown that deletion of exon three combined with type 1Δ causes BMKS. Hence, we can conclude that the variants identified in the probands of families 1 and 2 are the underlying causes of the clinical phenotype.

In the paper of Wieczorek *et al*^3^ the allele frequency of the promoter deletions in German control samples was estimated at 0.76%. A single homozygous type 1Δ deletion was identified in 3343 population-based German and South Asian samples. Unfortunately, data about the craniofacial phenotype in that individual were not available. In this study, we sequenced a cohort of 17 individuals with choanal atresia and also identified a homozygous type 1Δ deletion (family 3). Although the predicted frequency of homozygous type 1Δ deletions was estimated at 1:17 300, which seemed to exclude this genotype as a cause for BMKS, Wieczorek *et al*^3^ stated that this genotype might lead to a mild phenotype. It seems likely that the isolated bilateral choanal atresia identified in the proband of family 3 might be part of this mild phenotype, especially since choanal atresia is one of the major anomalies of BMKS. As isolated choanal atresia is a feature of the *Tbx22* knock-out mouse,^16^ the individuals with isolated choanal atresia were screened for mutations in *TBX22*, but causative variants were not found.

In family 4, homozygosity for the pathogenic promoter variant type 2Δ was found to segregate in the affected individuals. Wieczorek *et al*^3^ also found a homozygous type 2Δ case in an individual with true BMKS and showed that promoter activity of a construct containing type 2Δ was reduced by 72% compared to wild-type, indicating a stronger reduction of *TXNL4A* expression than type 1Δ. However, individuals with the type 2Δ (family 4) seem to be less severely affected than the compound heterozygotes; only choanal atresia, hypodontia and some minor anomalies were seen (Table 2). Further screening of family 4 identified eight members who were heterozygous for type 2Δ, and interestingly minor anomalies were present in two of them (Figure 1 and Table 2). Our data support the proposed dosage-specific effect.^3, 5^

Wieczorek *et al*^3^ identified causative variants in nine out of 11 families, compared to two out of 18 in this study. This may be attributed to the fact that in this work individuals were included with only mild features of BMKS and may overlap with other conditions such as bilateral craniofacial microsomia, CHARGE phenotype, and asymmetrical Treacher-Collins like phenotype. Recently, it has been shown that ribosomopathies, such as Treacher Collins syndrome, and spliceosomopathies can have similar craniofacial phenotypes.^5^ In contrast to CHARGE and Treacher Collins syndrome, individuals with BMKS tend to have a normal intellectual development, however, there is otherwise significant clinical overlap. Therefore, we suggest it will be important to test for mutations in *TXNL4A* if the clinical phenotype is indicative of BMKS, but also if the clinical phenotype is indicative of these other conditions should their appropriate gene tests report negative.

## Conclusion

We describe the finding of the splice site variant c.258-2A>G, combined with promoter deletion type 1Δ, in *TXNL4A* as the genetic cause of BMKS in two unrelated individuals. Homozygosity for the type 1Δ deletion was identified in a third individual with isolated choanal atresia. Homozygosity for the type 2Δ deletion was identified in a family with choanal atresia and other minor anomalies. These results confirm that variants affecting function of *TXNL4A* are the cause of BMKS and possibly a cause of isolated choanal atresia, underlining the tissue-specific nature of craniofacial disorders caused by spliceosomal defects.^5^

## Figures and Tables

**Table 1:** Clinical phenotype of individuals with causative variants using the features mentioned by Wieczorek *et al* ^3^ that are associated with BMKS

*Clinical phenotype of individuals with causative variants*					
*ID in pedigree*	*III.2 family 1*	*III.1 family 2*	*III.1 family 3*	*V.4 family 4*	*V.6 family 4*
Gender	Female	Male	Male	Male	Female
Age at examination	37 years			7 years	
Genetic testing	*TCOF1* *CHD7* SNP array WGS MLPA	Affymetrix 750 K microarray *TXNL4A* MLPA	Affymetrix 750 K microarray -> 18p11.32q23 (136,300-77,997,592) hmz uniparental disomy for chromosome 18 *TXNL4A* MLPA	Karyo *TXNL4A* MLPA	*TXNL4A* MLPA
Variant carried by father	c.258-2A>G, (p.?)	hg19 chr18:g.77748581_77748614del (type 1Δ)	hg19 chr18:g.77748581_77748614del (type 1Δ)	hg19 chr18: g.77748604_77748637del (type 2Δ)	hg19 chr18: g.77748604_77748637del(type 2Δ)
Variant carried by mother	hg19 chr18:g.77748581_77748614del (type 1Δ)	c.258-2A>G, (p.?)	hg19 chr18:g.77748581_77748614del (type 1Δ)	hg19 chr18: g.77748604_77748637del(type 2Δ)	hg19 chr18: g.77748604_77748637del(type 2Δ)
Positive family history	Y father scleral show	N	N	Y	Y
Normal pregnancy	Y	Polyhydramnios	IVF ICSI Failure to progress	Polyhydramnios	Polyhydramnios
Asymmetry of the face	Y			N	N
Hypertelorism	N			N	N
Short palpebral fissures	Y			N	N
Defect of lower eyelids	Y	Y (B)		N	N
Aplasia of puncta lacrimalis	Y (L)				
Prominent nasal bridge	Y	Y		Y	Y
Short philtrum	N			Y	Y
Thin lips	Y			Y	N
Cleft lip or palate	N	Y (L)		N	N
Bifid uvula	N			N	N
Bilateral choanal atresia/ stenosis	Bilateral (bony)	Bilateral	Bilateral (bony)	Bilateral (bony)	Bilateral
Prominent ears	Y			Y	Y
Preauricular tags	Y (R)	Y (R)			
Maxillary hypoplasia	Y (R)			Y	Y
Micrognathia	Y (R)	Y		N	
Cardiac defect	N	Asymptomatic ASD and VSD			
Hearing loss	N			N	N
Normal psychomotor development	Y	Y		Y	Y
Short stature	Y			N	N
Other	Hypoplasia infra-orbital rim (R) Upslanting palpebral fissures Eyelashes were longer laterally than medially Choroid coloboma (L) Microstrabismus Amblyopia	Dimple on the cheek Hypermetropia		Downslanting palpebral fissures Prognathism High arched narrow palate Absence of the upper and lower deciduous premolars Hypodontia of four permanent premolars	Cowlick Downslanting palpebral fissures Prognathism Dental malocclusion Narrow palate Unilateral absence of the permanent upper lateral incisor

Abbreviations: ASD, atrial septal defect; B, bilateral; Hom, homozygous; ICSI, intracytoplasmic sperm injection; IVF, *in vitro* fertilization; Karyo, karyotype; L, left; M, maternal; N, no; P, paternal; R, right; VSD, ventricular septal defect; WGS, whole-genome sequencing; Y, yes. Blank entries indicate that information was not available.

**Table 2:** Overview of testing done for 41 affected and 19 unaffected individuals

*Family*	*Sample*	*Gender*	*Genetic testing*	*Variants identified*	*MLPA*	*Clinical features*	*BMKS/isolated choanal atresia*
Family 1	III.2	F	*TCOF1*, *CHD7*, SNP array, WGS	hg19 chr18:g.77748581_77748614del (1Δ) (M), c.258-2A>G, (p.?) (P)	Y	Normal pregnancy, facial asymmetry, short palpebral fissures, defect of lower eyelids, aplasia of puncta lacrimalis L, hypoplasia infra-orbital rim R, upslanting palpebral fissures, longer eyelashes laterally than medially, choroid coloboma L, microstrabismus, amblyopia bilateral choanal atresia, preauricular tag R, maxillary hypoplasia R, micrognathia R and normal psychomotor development	BMKS
	II.1	M	WGS	c.258-2A>G, (p.?)	Y	Scleral show	
	II.2	F	WGS	hg19 chr18:g.77748581_77748614del (1Δ)	Y		
	Uncle	M	*TXNL4A*		Y		
	Husband	M	*TXNL4A*		Y		
Family 2	III.1	M	Affymetrix 750 K microarray, *TXNL4A*	hg19 chr18:g.77748581_77748614del (1Δ) (P), c.258-2A>G, (p.?) (M)	Y	Polyhydramnios, bilateral defect of lower eyelids, hypermetropia, prominent nasal bridge, cleft lip and palate L, bilateral choanal atresia, preauricular tag R, micrognathia, normal psychomotor development, dimple on the cheek, asymptomatic ASD and VSD	BMKS
	II.2	F	*TXNL4A*	c.258-2A>G, (p.?)	N		
	II.1	M	*TXNL4A*	hg19 chr18:g.77748581_77748614del (1Δ) (het)	N		
Family 3	III.1	M	Affymetrix 750 K microarray -> isodisomy of chr18 *TXNL4A*	hg19 chr18:g.77748581_77748614del (1Δ) (hom)	Y	IVF ICSI, failure to progress and bilateral choanal atresia	Choanal atresia
	II.2	F	*TXNL4A*	hg19 chr18:g.77748581_77748614del (1Δ) (het)	N		
	II.1	M	*TXNL4A*	hg19 chr18:g.77748581_77748614del (1Δ) (het)	N		
Family 4	V.4	M	*TXNL4A*, *TBX22*	hg19 chr18: g.77748604_77748637del (2Δ) (hom)	Y	Polyhydramnios, downslanting palpebral fissures, prominent nasal bridge, bilateral choanal atresia, maxillary hypoplasia, prognathism, high arched narrow palate, absence of the upper and lower deciduous premolars, hypodontia of four permanent premolars and normal psychomotor development	Choanal atresia
	V.6	F	Karyo, *TXNL4A*, *TBX22*	c.-245_-212del34, (p.?) (2Δ) (hom)	Y	Polyhydramnios, prominent nasal bridge, bilateral choanal atresia, maxillary hypoplasia, cowlick, downslanting palpebral fissures, prognathism, dental malocclusion, narrow palate and unilateral absence of the permanent upper lateral incisor	Choanal atresia
	III.1	F	*TXNL4A*		N		
	III.2	F	*TXNL4A*	hg19 chr18: g.77748604_77748637del (2Δ) (het)	N		
	IV.1	F	*TXNL4A*		N		
	IV.2	M	*TXNL4A*	hg19 chr18: g.77748604_77748637del (2Δ) (het)	N	Prognathism	
	IV.3	F	*TXNL4A*		N		
	IV.4	M	*TXNL4A*	hg19 chr18: g.77748604_77748637del (2Δ) (het)	N		
	IV.5	M	*TXNL4A*	hg19 chr18: g.77748604_77748637del (2Δ) (het)	N	Prognathism	
	IV.6	M	*TXNL4A*	hg19 chr18: g.77748604_77748637del (2Δ) (het)	N		
	IV.7	F	*TXNL4A*	hg19 chr18: g.77748604_77748637del (2Δ) (het)	N		
	V.1	F	*TXNL4A*		N		
	V.2	M	*TXNL4A*		N		
	V.3	M	*TXNL4A*		N		
	V.5	M	*TXNL4A*	hg19 chr18: g.77748604_77748637del (2Δ) (het)	N		
	V.7	M	*TXNL4A*	hg19 chr18: g.77748604_77748637del (2Δ) (het)	N		
Family 5	II.1	M	WGS, *TXNL4A*		Y	Ptosis, shallow orbits, prominent beaked nose and micrognathia	BMKS
	I.2 (mother)	F	WGS, *TXNL4A*		Y		
	6	M	WGS, *TXNL4A, TCOF1, POLR1D, POLR1C, POLR1A,* array		Y	Polyhydramnios, premature, respiratory distress, macroglossia, micrognathia, retrognathia, cleft palate, bilateral microtia, preauricular fistula, short downslanting palpebral fissures, shallow orbits and hearing loss	BMKS
	7	M	*TXNL4A*, array, *TCOF1*, *SALL1*, *POLR1C*, *POLR1D*		Y	Small for gestational age, conductive hearing loss, bilateral ear tags, downslanting palpebral fissures, hypertelorism, micrognathia, perianal tag and patent foramen ovale	BMKS
	8	F	*TXNL4A*, *CHD7*		Y	Bilateral choanal atresia	Choanal atresia
	9	F	*TXNL4A*		Y	Bilateral Tessier 7 cleft, dental crowding, bilateral ear tags, hearing loss based on absent cochlear nerves, coloboma of the left papill, retina and pupil, amblyopia, esophageal atresia with tracheoesophageal fistula, hemivertebrae, hypoplastic thumbs L>R, bilateral hemifacial macrosomia, hypotonia, OSA and mild PMR	BMKS
	10	M	Karyo, *TXNL4A*		Y	Micrognathia, retrognathia, narrow maxilla, bilateral ear tags, hearing loss, epibulbar dermoid (R), dental crowding and OSA	BMKS
	11	F	All TCS genes, WES, BOR genes		Y	Asymmetry of the face, hearing loss, normal stature, dysplastic ears, amblyopia and aberrant facial nerve	BMKS
	12	F	*TCOF1*, *POLR1C*, *POLR1D*, WES, *TXNL4A*	deletion of *POLR1D*	Y	Asymmetry of the face, hypertelorism, short palpebral fissures, prominent nasal bridge, cleft lip and palate, microtia, maxillary hypoplasia, micrognathia, hearing loss, normal psychomotor development and short stature	BMKS
	13	M	all TCS genes, *TXNL4A*		Y	Unilateral cleft lip and palate, maxillary hypoplasia, micrognathia and normal psychomotor development	BMKS
	14	M	*TCOF1*, array, BOR syndrome genes, *TXNL4A*		Y	Renal cysts and microtia (unilateral)	BMKS
	15	F	*TCOF1*, SNP array, *POLR1C*, *POLR1D*, *TXNL4A*		Y	Bilateral preauricular tags, bilateral Tessier 7 cleft, microtia R and anal atresia	BMKS
	16	M	*TCOF1*, *TXNL4A*		Y	Maxillary hypoplasia, micrognathia, microtia R and hypoplasia zygomata (mild)	BMKS
	17	M	*TCOF1*, *TXNL4A*		Y	Maxillary hypoplasia, micrognathia and unilateral hearing loss	BMKS
	18		*TXNL4A*, *TBX22*		Y	Hypertelorism, hypoplastic uvula, right sided choanal atresia, Mum Carbimazole treatment, blue eyes and ear pit (cochlear implant)	Choanal atresia
	19		*TXNL4A*, *TBX22*		Y	Choanal atresia R	Choanal atresia
	20		*TXNL4A*, *TBX22*		Y	Choanal atresia B	Choanal atresia
	21		*TXNL4A*, *TBX22*		Y	Choanal atresia R	Choanal atresia
	22		*TXNL4A*, *TBX22*		Y	Bifid uvula, choanal atresia R, ASD	Choanal atresia
	23		*TXNL4A*, *TBX22*		Y	Choanal atresia R	Choanal atresia
	24		*TXNL4A*, *TBX22*		N	Choanal atresia R	Choanal atresia
	25		*TXNL4A*, *TBX22*		N		Choanal atresia
	26		*CHD7*, *TXNL4A*, *TBX22*	R157X in *CHD7*	N	CHARGE	Choanal atresia
	27		*TXNL4A*, *TBX22*		N		Choanal atresia
	28		*TXNL4A*, *TBX22*		N		Choanal atresia
	29		*TXNL4A*, *TBX22*		N		Choanal atresia
	30		*TXNL4A*, *TBX22*		N		Choanal atresia
	31		*TXNL4A*, *TBX22*		N	Choanal atresia L, ID and syngnathia	Choanal atresia
	32		*TXNL4A*		N		BMKS
	33	F	*TXNL4A*		N		BMKS
	34	M	*TXNL4A*		N		BMKS
	35	M	*TXNL4A*		N		BMKS
	36	M	*TXNL4A*		N		BMKS

Abbreviations: ASD, arial septal defect; B, bilateral; f, female; Hom, homozygous; ICSI, intracytoplasmic sperm injection; ID, intellectual disability; IVF, *in vitro* fertilization; karyo, karyogram; L, left; m, male; M, maternal; N, no; OSA, obstructive sleep apnea; P, paternal; PMR, psychomotor retardation; R, right; VSD, ventricular septal defect; WES, whole-exome sequencing; WGS, whole-genome sequencing; Y, yes.
