# Supplementary material for: Identification of causative variants in TXNL4A in Burn-McKeown syndrome and isolated choanal atresia
Source: Eur J Hum Genet. 2017 Jul 26;25(10):1126–33. doi: 10.1038/ejhg.2017.107 (PMC5602009; doi:10.1038/ejhg.2017.107)
Supplement: Supplementary Table 1 [file ejhg2017107x1.docx]

| Primers used for PCR and Sanger sequence analysis | | | | |
| --- | --- | --- | --- | --- |
|  | Amplicon | 5'→ 3' | | Product size (bp) |
|  |  | Forward | Reverse |  |
| DNA analysis | Promoter2 F/R | gcctgatggtgacattcctc | ctgacggcatgtgcgtatag | 457 |
|  | Promoter1 F/R | ctccgacagctgctatgacg | gaggatggcctggtccac | 400 |
|  | Exon1 F/R | gggccggggagtgag | ggaggaatcgcctgaacg | 377 |
|  | Exon2 F/R | gccctgatccacacaaattc | ctgtacaaccaacttccttcaaaa | 318 |
|  | Exon3 F/R | gcttgtcacagtgggaaaaat | gccctggagcttcctgtat | 366 |
| cDNA analysis | F1/R3 | gatcctacgtgcatgaagatggacg | agtagtccttgggggacacca | 307 |
|  | F2/F3 | aattttgcagttatttatcttgtggatattacag | agtagtccttgggggacacca | 250 |
|  | F2/In2R1 | cttgtggatattacagaagtgcctgacttc | ctaacgcataccctatacaacccacc | 999 |
|  | F2/In2R2 | cttgtggatattacagaagtgcctgacttc | caaaagcaacacactcgggatatttagc | 1902 |
|  | F2/In2R3 | cttgtggatattacagaagtgcctgacttc | gatgcaggagaatacaggaagcagc | 3679 |
|  | F2/R5 | cttgtggatattacagaagtgcctgacttc | aggctcacaggataaacaccttcgtttta | 535 |
|  | F2/R8 | cttgtggatattacagaagtgcctgacttc | cagctggtgtcactacgaaaagg | 847 |
|  | F2/R9 | cttgtggatattacagaagtgcctgacttc | ctaactatatgaactgaagggccatggc | 1160 |
|  | F4/R5 | cacctctgagatggaattgataacatgg | aggctcacaggataaacaccttcgtttta | 178 |
|  | F4/R8 | cacctctgagatggaattgataacatgg | cagctggtgtcactacgaaaagg | 490 |
|  | F4/R9 | cacctctgagatggaattgataacatgg | ctaactatatgaactgaagggccatggc | 803 |
| Digest | F10/R11 | cagagatgtgtgctctcacagctttgcag**a**cggggg | ccaacacagctggtgtcactacgaaaaggg | 249 |
| PGM | F12/R13 | cgctcttccgatctctggcgttttgcatactgggttggttt | tgctcttccgatctgacgtatccacacaccaaggaactgaa | 225 |
|  | F14/R15 | ccatctcatccctgcgtgtctccgactcagagcactgtagcgctcttccgatctctg | cctctctatgggcagtcggtgattgctcttccgatctgac | 225 |

Supplementary Table 1. Primers used for PCR and dideoxy-sequence analysis. Primer sequence from 5' to 3'.
